# Vertical changes in the hard tissues after space closure by miniscrew sliding mechanics: a three-dimensional modality analysis

**DOI:** 10.1186/s13005-023-00388-9

**Published:** 2023-12-04

**Authors:** Hong Su, Zimeng Zhuang, Bing Han, Tianmin Xu, Gui Chen

**Affiliations:** 1 First Clinical Division, Peking University School and Hospital of Stomatology, Beijing, 100034 China; 2 National Center of Stomatology & National Clinical Research Center for Oral Diseases & National Engineering Laboratory for Digital and Material Technology of Stomatology & Beijing Key Laboratory for Digital Stomatology & Research Center of Engineering and Technology for Computerized Dentistry Ministry of Health & NMPA Key Laboratory for Dental Materials, Beijing, 100081 China; 3 Department of Orthodontics, Cranial-Facial Growth and Development Center, Peking University School and Hospital of Stomatology, Beijing, 100081 China

**Keywords:** Miniscrew, Sliding mechanics, Space closure, Vertical change

## Abstract

**Objectives:**

This study aimed to investigate vertical changes in the maxillary central incisor and the maxillary first molar, along with alterations in the mandibular plane angle during space closure using miniscrew sliding mechanics.

**Methods:**

Twenty adult patients treated at Peking University Hospital of Stomatology between 2008 and 2013 were included. Digital dental models and craniofacial cone-beam computed tomography (CBCT) scans were obtained at the start of treatment (T0) and immediately after space closure (T1). Stable miniscrews were used for superimposing maxillary digital dental models (T0 and T1), and vertical changes in the maxillary first molar and the maxillary central incisor were measured. Three-dimensional changes in the mandibular plane were assessed through CBCT superimposition.

**Results:**

The maxillary central incisor exhibited an average extrusion of 2.56 ± 0.18 mm, while the maxillary first molar showed an average intrusion of 1.25 ± 1.11 mm with a distal movement of 0.97 ± 0.99 mm. Additionally, the mandibular plane angle decreased by an average of 0.83 ± 1.65°. All three indices exhibited statistically significant differences.

**Conclusion:**

During space closure using the miniscrew sliding technique, significant changes occurred in both the sagittal and vertical dimensions of the upper dentition. This included extrusion of the maxillary central incisors, intrusion of the maxillary first molars, and a slight counterclockwise rotation of the mandibular plane.

## Introduction

Miniscrews are a common method for controlling tooth anchorage in clinical practice [1–3]. They offer advantages such as a small volume, independent patient compliance, and the provision of absolute anchorage in all three dimensions [4]. Their greatest superiority lies in the vertical direction. In certain conditions, such as treating overgrown molars, addressing posterior scissor bites, and correcting open bites by intruding posterior teeth on both sides, miniscrews outperform traditional anchorages [5–7]. In the lateral direction, miniscrew-supported skeletal expanders can prevent undesirable tooth movement, which can be detrimental to periodontal health when compared to traditional tooth-borne expanders [8, 9]. Additionally, miniscrews can effectively open mid-palatal sutures in most adult patients with developed sutures, benefiting those with skeletal transverse maxillary deficiencies [10]. The most common clinical applications of miniscrews involve enhancing sagittal molar anchorage and retracting anterior teeth to reduce protrusion. Studies indicate that during incisor retraction, miniscrew anchorage can reduce mesial molar movement by approximately 2 mm compared to traditional anchorage [11]. Researchers have investigated both incisor retraction and mesial molar movement, providing clear insights into sagittal tooth changes [12, 13].

Understanding vertical tooth changes is more challenging due to their complex nature. Lee et al. analyzed anterior teeth retraction using the sliding method and noted that vertical changes depended on miniscrew placement [14]. Kawamura et al. took a comprehensive approach by employing three-dimensional finite element analysis to study how different arch wire sizes affect incisor and molar movement during miniscrew sliding technique for space closure. They observed rotation of the upper dentition after gap closure, with extrusion of upper central incisors and intrusion of molars [15].

In orthodontics, molar intrusion is crucial for controlling vertical dimensions and mandibular position, especially in high angle cases [7, 16, 17]. If the miniscrew sliding technique for space closure can not only enhance molar sagittal anchorage but also adjust molar vertical position to control mandibular vertical growth in teenagers or improve the facial profile of skeletal class II patients with high angles, it would have significant clinical implications [18].

This study evaluates the vertical changes in the maxillary first molar and central incisor following space closure using miniscrew sliding mechanics. It also examines changes in the mandibular plane using three-dimensional digital methods, which have been demonstrated to be superior to two-dimensional methods [19].

## Method and materials

### Participants

The samples used in this study were collected from 2008 to 2013 at the Peking University School and Hospital of Stomatology. Detailed information regarding their recruitment process, miniscrew implantation, and orthodontic treatment has been previously published [20, 21]. The study included 20 patients (14 females and 6 males) aged 21–41 years, with a mean age of 24 years. The sample comprised 13 Angle Class I and 7 Class II malocclusions, including 11 skeletal Class I and 9 skeletal Class II malocclusions. The average overbite (OB) was 2.9 mm, with one case presenting with an open bite, and the average overjet was 4.1 mm. Ethical approval for this study was obtained from the Biomedical Ethics Committee of Peking University (approval number: IRB00001052-09010). Informed consent was obtained from all participants.

To clearly investigate the impact of miniscrews in the process of space closure, this study specifically focused on patients with mild crowding who primarily sought relief from protrusion. Individuals with craniofacial growth and development issues or pathological conditions were excluded to minimize the influence of confounding factors. The inclusion criteria were as follows: (1) age > 18 years, (2) significant protrusion of the upper teeth, (3) extraction of the maxillary first premolar, (4) utilization of the miniscrew sliding technique for space closure, and (5) overall good health without chronic diseases or disabilities. Exclusion criteria were as follows: (1) upper dentition crowding > 4 mm, (2) congenital loss of maxillary permanent teeth (except for the first premolars and third molars), (3) history of cranial or facial trauma, and (4) cleft lip and palate or syndromic conditions.

### Treatment procedure

During the treatment, we used two types of self-drilling miniscrews (diameter: 1.6 mm, length: 11 mm; Ci Bei Corp., Zhejiang, China). Two of these miniscrews were loaded and inserted into the buccal interradicular space between the maxillary second premolar and the first molar on either side. This location offered a clear operating field and minimized the risk of injuring tooth roots. Four additional miniscrews were placed but left unloaded. The unloaded miniscrews were inserted between the lateral incisor and canine in the anterior region and between the first and second molars in the posterior region.

All patients underwent treatment with McLaughlin-Bennett-Trevisi (MBT™) straight wire appliances. Nickel-titanium wire was used in the initial aligning and leveling stage. For space closure using the miniscrew sliding technique, we employed a 0.019 inch × 0.025 inch stainless steel wire along with free traction hooks (4 mm in length) placed between the maxillary lateral incisors and canines. This wire size was selected for its adequate strength to resist arch wire deformation. A traction force of approximately 150 g per side was applied using powerchains for space closure. Aside from the miniscrews, no additional devices were used for anchorage enhancement. On average, the duration of anterior teeth retraction using miniscrews was 11.8 months.

### Data acquisition and measurement

Maxillary impressions were taken at two key time points: before treatment (T0) and after space closure using the miniscrew sliding technique (T1). These impressions were captured using silicone rubber and subsequently used to create plaster models. To digitize the maxillary models, we employed a 3D laser scanner (3Shape R700, 3Shape A/S, Copenhagen, Denmark) with a scanning accuracy of ± 0.02 mm. For Cone Beam Computed Tomography (CBCT) scans, the same technician and machine (DCT Pro, Vatech Co., Yongin-Si, Korea) were used at both T0 and T1. The scan parameters were as follows: a scanning field of 20 cm × 19 cm, a tube voltage of 90 kV, a tube current of 7 mA, and a scanning duration of 15 s. During the scans, patients were positioned in their natural head posture with the mandible in the intercuspal position.

#### Three-dimensional displacement of maxillary first molars and maxillary central incisors

The three-dimensional displacement of the first molars and maxillary central incisors was measured on digital dental models using Rapidform2006 (INUS Technology Inc., Seoul, Korea). The measurements and their definitions were shown in Table 1.

**Table 1:** Variables and their definitions

Variable	Definition
6-X	Sagittal movement of the maxillary first molar
6-Y	Vertical movement of the maxillary first molar
6-Z	Lateral movement of the maxillary first molar
6-Total	Total movement of the maxillary first molar
1-X	Sagittal movement of the maxillary central incisor
1-Y	Vertical movement of the maxillary central incisor
1-Z	Lateral movement of the maxillary central incisor
1-Total	Total movement of the maxillary central incisor
ΔMP	Angular variation in the mandibular plane

##### Maxillary digital dental model superimposition

**Fig. 1:**
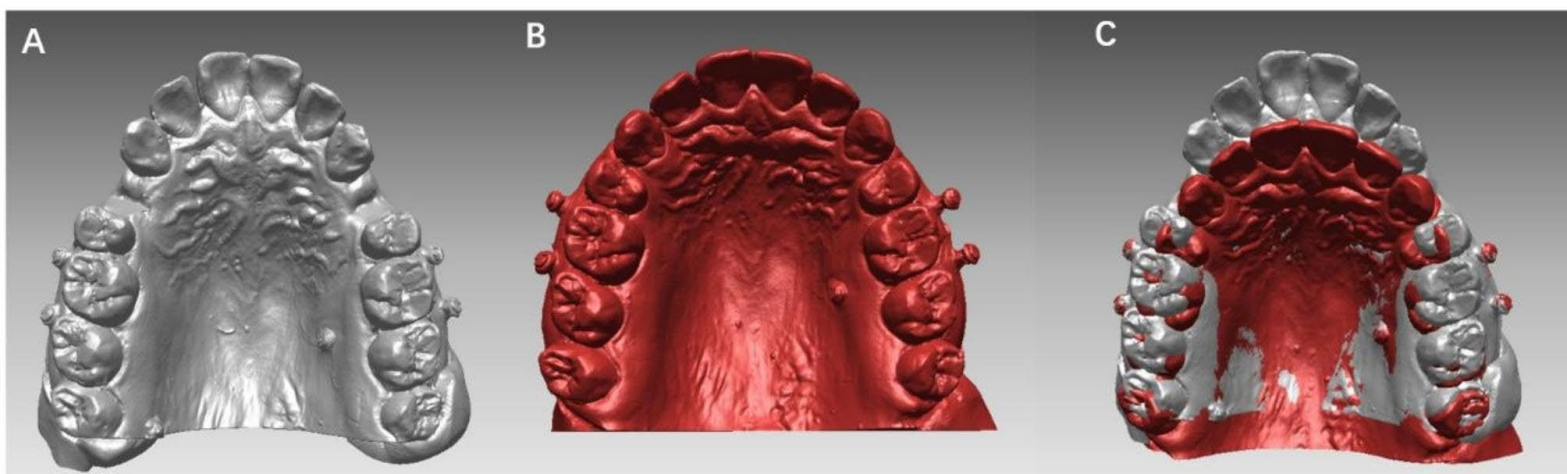
(**a**) Maxillary digital dental model with 6 miniscrews before treatment (T0); (**b**) Maxillary digital dental model with 6 miniscrews after space closure (T1); (**c**) The result of miniscrew superimposition. All 6 miniscrews overlapped well and showed a perfect superimposition result

Maxillary digital dental models at T0 and T1 were superimposed using stable miniscrews (Fig. 1).

##### Landmark transfer

**Fig. 2:**
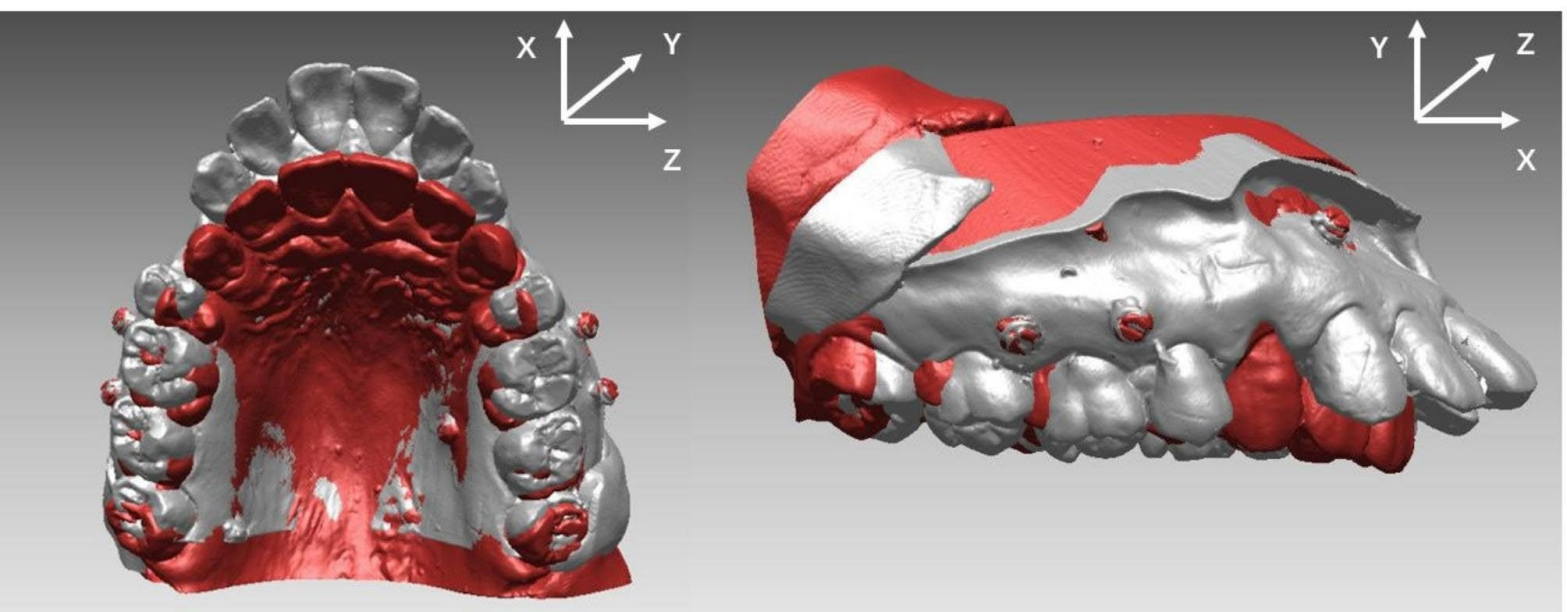
The three-dimensional local reference coordinates used in this study

Mesiobuccal cusps of the maxillary first molars and midpoints of the central incisor edges were marked on the T0 digital models. The landmarks were transferred from the T0 model to the T1 model through individual tooth registration. Errors from tooth landmark identification were excluded (Fig. 2).

##### Local reference coordinate establishment

The occlusal plane, based on the mesial buccal cusps of the maxillary first molar and midpoints of the central incisor edges, was defined as the transverse plane. Two points were marked on the palatal suture as points A and B, and projected onto the occlusal plane to obtain points A’ and B’. We set point B’ as the origin, B’-A’ as the x-axis, and B’-B as the z-axis to establish a three-dimensional reference coordinate.

##### Tooth movement measurement

Using the local reference coordinate system, we assessed the three-dimensional displacement changes in the maxillary first molars and maxillary central incisors using specialized software. All measurements represent the averages of replicates from independently conducted landmark location and digital cast superimposition procedures. The changes observed on the right and left sides were averaged to ensure accuracy and consistency of assessment.

#### Vertical changes in the mandibular plane

**Fig. 3:**
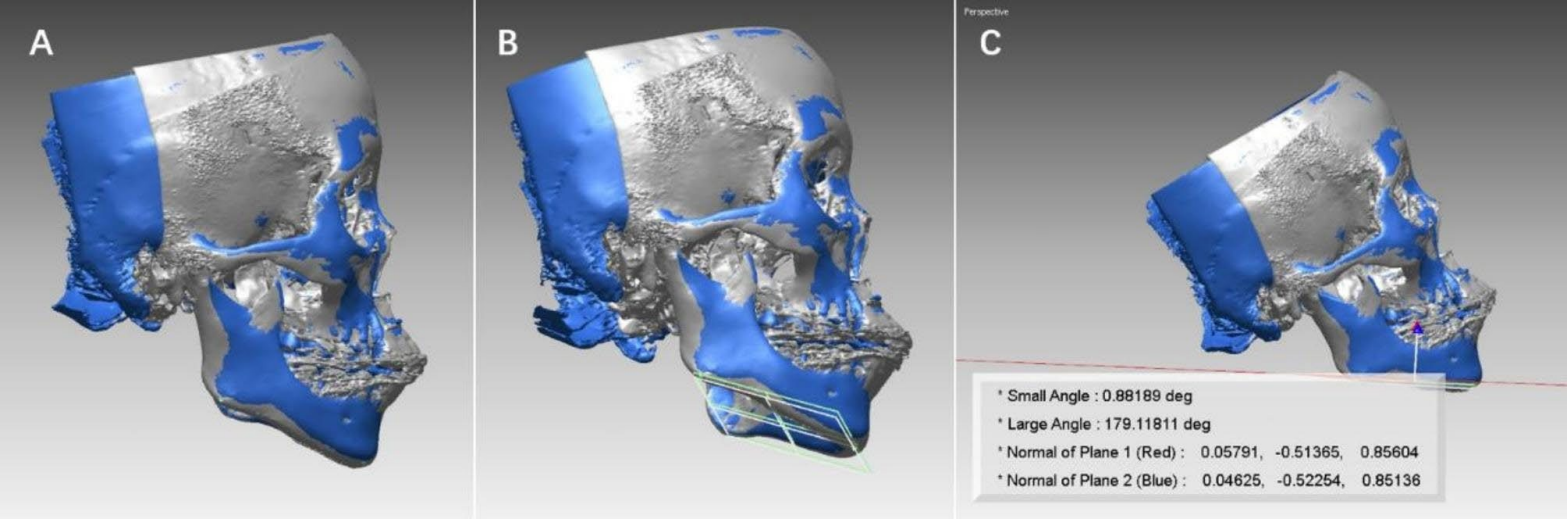
(**a**) Stable areas, such as the cranial base, frontal bone, and cheekbones were used for superimposition at T0 (gray) and T1 (blue); (**b**) Mandibular planes are shown in white (T0) and green (T1) colors in the T0 model (gray) and T1 model (blue); (**c**) Rapidform calculation of the angle between the two planes, which indicated the change in the mandibular plane, is shown

**Fig. 4:**
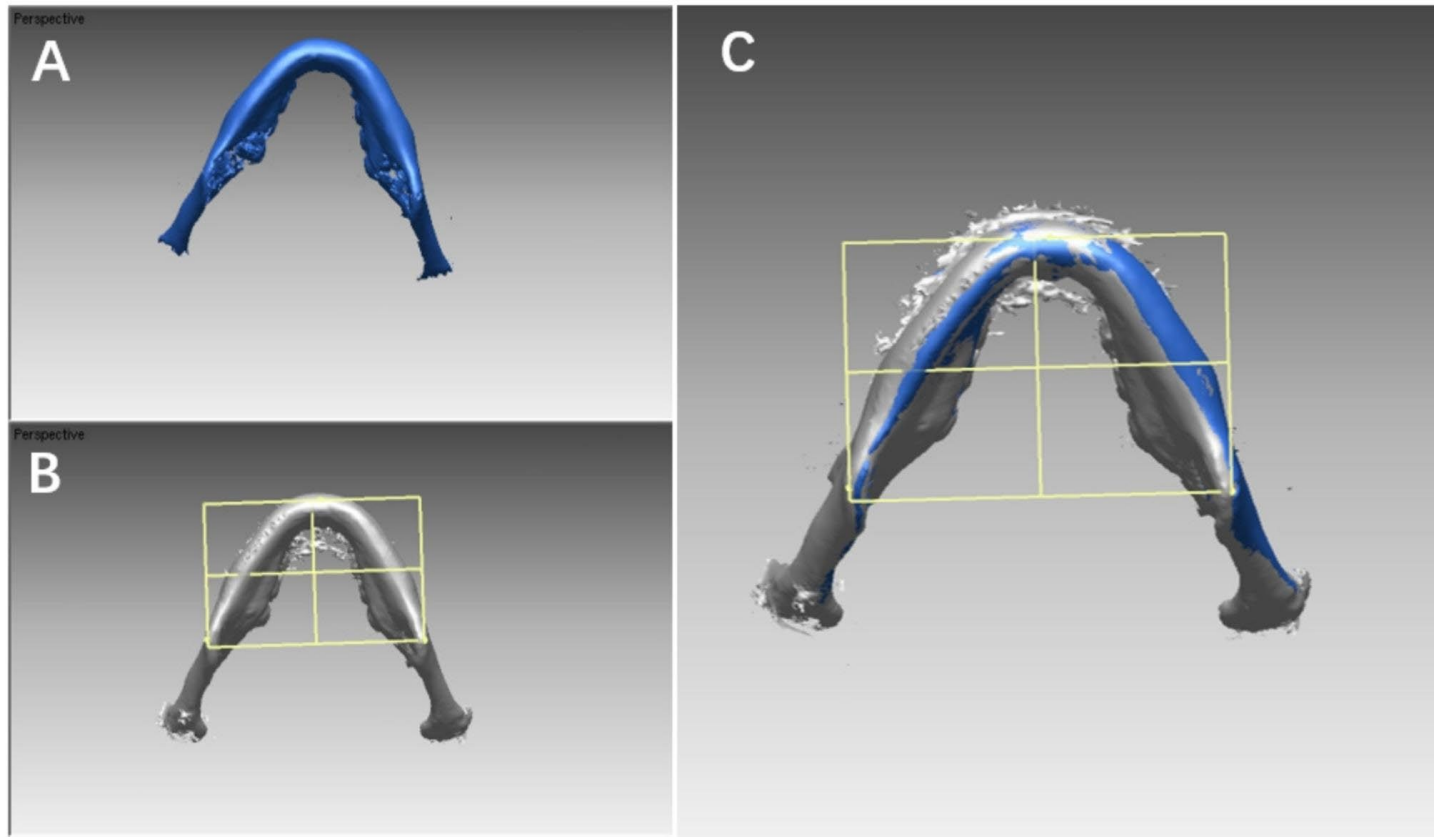
(**a**) Mandible at T1; (**b**) Mandible and mandibular plane at T0 that were bonded with each other; (**c**) By mandibular superimposition, the mandibular plane on the T0 model was transferred to the T1 model, which excluded the errors from tooth landmark identification at different timepoints

To measure changes in the mandibular plane, we utilized CBCT scans and defined the mandibular plane in three-dimensional space as a plane formed by the gonion (Go) and menton (Me) points on either side. Initially, the patient’s computed tomography (CT) data were saved in Digital Imaging and Communication in Medicine (DICOM) format and managed using an interactive medical image control system (MIMICS 10.0, Materialise, Leuven, Belgium). This system was employed to construct a three-dimensional surface model of craniofacial hard tissues. The resulting data were then exported in STL format and imported into Rapidform 2006. Stable anatomical regions, such as the cranial base, frontal bone, and cheekbones, were used as reference points for superimposition. To quantify the change in the mandibular plane, we measured the angle between the mandibular planes at T1 and T2, denoted as ΔMP (Fig. 3). We adopted the landmark transfer technique, specifically referencing the plane transfer technique where a reference plane is established using three marked points. Three landmarks were located on the mandibular surface of the model at T0 and then transferred to the surface model at T1 through the superimposition of stable areas within the mandible (Fig. 4). Two observers, both possessing similar clinical experience, underwent simultaneous calibration training encompassing various aspects, including software operation, landmark definition, selection of stable regions, and other relevant procedures. Subsequently, the measurements were carried out twice by the trained observers following the same standardized procedure, and the results were averaged for accuracy and consistency.

### Statistical analysis

Data analysis was performed using SPSS Statistics (version 23.0, IBM Corp., Armonk, NY). P-values < 0.05 were considered statistically significant. The statistical hypothesis was that maxillary miniscrew sliding technique would allow the maintenance of the original sagittal and vertical factors, which could be quantified through changes in upper first molars, central incisors, and the mandibular plane. Based on this statistical hypothesis, one-sample t-tests were conducted to compare changes observed between T0 and T1 with a reference value of 0. This allowed us to assess alterations during the period of space closure using the maxillary miniscrew sliding technique. A statistically significant result indicated that a vertical or sagittal change occurred during space closure with the maxillary miniscrew. Conversely, a lack of statistical significance indicated that the positions of the upper first molars, central incisors, and mandibular plane remained stable throughout the treatment. To evaluate the reliability of measurements obtained from digital dental models and the 3D mandibular plane change measurement method, we calculated intraclass correlation coefficients (ICCs) between the two observers. ICC values > 0.9 were considered to indicate excellent reliability in the measurements.

## Results

The three-dimensional movements of maxillary first permanent molars, maxillary central incisors, and the remaining components are shown in Table 2. During space closure by the miniscrew sliding technique, the maxillary first molar was intruded by 1.29 ± 0.88 mm, while the maxillary central incisor was extruded by 2.68 ± 1.77 mm, both indicating statistically significant changes. Furthermore, the maxillary first molar intruded with a distal movement of 0.97 ± 0.99 mm, and this change was also statistically significant. Additionally, the mandibular plane rotated counterclockwise by an angle of 0.83 ± 1.65°.

**Table 2:** Changes in the first molar, central incisor, and mandibular plane during space closure using the maxillary miniscrew sliding technique (N = 20)

Variable	Mean	Standard deviation	T	P value
6-X (mm)	0.97	0.99	4.345	0.000
6-Y (mm)	1.29	0.88	6.528	0.000
6-Z (mm)	−0.37	1.12	−1.478	0.156
6-Total (mm)	2.24	0.75	13.318	0.000
1-X (mm)	7.12	2.35	13.548	0.000
1-Y (mm)	−2.68	1.77	−6.764	0.000
1-Z (mm)	0.55	2.16	1.133	0.271
1-Total (mm)	7.99	2.72	13.141	0.000
ΔMP (°)	0.83	1.65	−2.230	0.038

A positive value of X indicates distal movement, while a negative value indicates mesial movement. Positive values of Y indicate intrusion, while negative values indicate extrusion. A positive Z value indicates buccal movement and a negative value indicates lingual movement. A positive ΔMP value indicates counterclockwise rotation, whereas a negative value indicates clockwise rotation.

ICC values were 0.991 for digital dental model measurements and 0.905 for 3D mandibular plane change measurements.

## Discussion

The miniscrew sliding technique for space closure is a widely employed method for treating severe dental protrusions. This technique is particularly effective in maintaining the sagittal anchorage of molars, which allows for substantial retraction of anterior teeth and significantly improves patients’ lateral profiles. While primarily utilized for anterior teeth retraction, the miniscrews induce such pronounced sagittal changes that their vertical impact is often overlooked. Although several studies have reported the effects of miniscrews on anterior teeth retraction using two-dimensional cephalometric measurements [13, 22], a comprehensive three-dimensional analysis, especially regarding vertical changes, remains lacking.

In terms of methodology, this study utilized three-dimensional digital data and adopted three-dimensional measurement techniques, providing more comprehensive and accurate results compared to traditional two-dimensional lateral cephalograms [23–26]. Our sample included both loaded and unloaded miniscrews, which allowed for optimal superimposition and enhanced measurement accuracy. We initially superimposed digital maxillary dental models using stable miniscrews. Subsequently, we established a local coordinate system to break down the total vectors of tooth movement into three dimensions, a familiar concept for orthodontists. Tooth retraction primarily involves forward and backward movements along the occlusal plane, while extrusion and intrusion mainly occur perpendicular to the occlusal plane. Furthermore, we employed the landmark transfer technique in this study [27, 28]. Landmarks on the teeth at T0 were transferred to the T1 model through individual tooth registration. This process eliminated errors stemming from multiple landmark localization steps, enhancing measurement precision.

Regarding the measurement of the mandibular plane, we created a three-dimensional mandibular plane and calculated plane-to-plane angles for the first time. This approach was significantly more advanced and accurate than traditional methods. In traditional lateral cephalograms, the mandibular plane is represented as a line comprising two points (commonly Go and Me). Measuring changes in the mandibular plane involves placing two points on the lateral cephalogram films before and after treatment. However, differences in X-ray incident angles and patient head positions can introduce errors, especially in locating Go. Baumrind et al. [23] demonstrated that locating Go had a relatively high error rate, particularly when measuring subtle changes in the mandibular plane. In our study, we selected three points on the three-dimensional reconstructed mandible to construct an actual mandibular plane, which we then bonded to the mandible before treatment (T0). These points included Go on both sides and the pogonion (Po). We transferred the mandibular plane at T0 to the mandible at T1 using mandibular superimposition, focusing on stable structures within the mandible that excluded lower teeth, lower alveolar bone, and the condyle [29]. This approach avoided multiple landmark localization processes and improved the precision of mandibular plane change measurements. After superimposing these stable structures, which included the anterior skull base, we measured the angle between mandibular planes at T0 and T1 to assess mandibular rotation following anterior teeth retraction using the miniscrew anchorage sliding technique.

Our results revealed significant sagittal retraction of the maxillary central incisor’s incisal edge (mean value of 7.12 mm), consistent with previous findings [30]. Simultaneously, we observed distal movement of the mesiobuccal cusp of the maxillary first molar (average of 0.97 mm), which differed from outcomes observed with the MBT sliding method for space closure but aligned with the results of a study by Upadhyay [22]. The MBT technique initially employs canine laceback during alignment, causing mesial movement of the first molar. However, during space closure, anchored molars continue moving mesially when retracting six anterior teeth. In contrast, the miniscrew-anchorage sliding technique generates a distal movement force for maxillary molars through friction against the molar buccal tube canal. The magnitude of this friction force is associated with positive pressure, which results from the vertical pressure exerted by the arch wire on the lumen. The force applied to the miniscrew, generating torque to rotate anterior teeth, is also related to this pressure.

The results in the vertical direction showed a decrease in the mandibular plane angle by an average of 0.83 ± 1.65°, indicating a slight counterclockwise rotation of the mandible. These findings can be beneficial for the treatment of patients with skeletal Class II malocclusions and those with high angles. However, this study also revealed an average extrusion of 2.68 mm in the maxillary central incisor, which may be inconsistent with the intuitive perception of many orthodontists. Some orthodontists believe that the use of miniscrews for retraction should intrude the upper incisors, but this study suggests otherwise. In fact, when the line of action of the miniscrew retraction force does not pass through the center of resistance of the upper dentition, the force applied to the dentition can be divided into a force and a moment passing through the center of resistance, according to the principles of the equal effectiveness system. The combination of this force and torque determines how the dentition moves. This study’s findings align with those of Kojima et al., who analyzed the influence of different force directions on the entire dental arch using a three-dimensional finite element method [31]. They concluded that these effects may be related to the use of short traction hooks on anterior teeth, similar to those used in this study. Additionally, Lee et al. used miniscrews placed between the maxillary first premolar and the first molar, along with short traction hooks in the anterior area for retraction based on the sliding method. In their research, they found that upper incisors extruded by an average of 0.25 mm, which is similar to the results of this study [14]. Another study by Kawamura et al. used a three-dimensional finite element method to analyze the influence of different arch wire sizes on how incisors and molars moved when space was closed using the miniscrew-anchorage sliding technique. They found that a smaller arch wire created a larger clearance between arch wires and slots, resulting in greater elastic deformation of the arch wire. This led to upper incisors being more likely to incline lingually and extrude when a smaller arch wire was used. There was also a slight intrusion observed in the posterior teeth, which had a weak correlation with the size of the arch wire [15]. These findings are consistent with the results of this study.

The present study emphasizes the importance of taking into consideration the vertical changes induced by the miniscrew sliding technique. The extrusion of upper incisors and intrusion of upper molars observed in this study can be advantageous for treating cases with high angles and open bites from a vertical perspective. Orthodontists can utilize the techniques and devices described in this study to achieve desirable vertical changes when employing the miniscrew sliding technique. Furthermore, the results indicate that the miniscrew-anchorage sliding technique is a viable choice for space closure, particularly for patients with severe tooth protrusion. However, caution must be exercised when treating patients with gummy smiles or anterior deep overbites, as extrusion of the upper incisors during miniscrew-anchorage space closure may not be suitable.

It is also important to note that the current study had limitations, as there are various factors affecting tooth movement during space closure with the miniscrew-anchorage sliding technique, including miniscrew position, height of the traction hook, arch wire size and shape, and patient-specific factors (age, bone type, periodontal condition, etc.). The devices used in this study included 0.022 inch × 0.028 inch brackets and 0.019 inch × 0.025 inch stainless steel flat arches, with short traction hooks placed between the maxillary lateral incisors and canines. Miniscrews were positioned at the height of the mucogingival junction between the maxillary second premolars and first molars, and a traction force of approximately 150 g per side was applied between the miniscrews and the short traction hook. These specific parameters resulted in extrusion of the upper incisors, intrusion of the maxillary first molars, and a slight counterclockwise rotation of the mandibular plane. However, these findings may not be universally applicable to all clinical scenarios. Further research is needed to explore the mechanics and variables involved in the miniscrew sliding technique and its application in different situations, considering variations in the position of traction hooks and miniscrews, wire and bracket combinations, and patient age. Clinicians should carefully select the appropriate combination of devices and construct a tailored mechanical system to achieve the desired tooth movement based on the specific clinical needs of each patient.

## Conclusion

1. The miniscrew sliding technique effectively maintains molar anchorage in the sagittal direction, with some cases even showing distal movement of the maxillary first molars.
2. Under specific conditions, including miniscrew position, traction hook height, arch wire characteristics, and sample selection, space closure using the miniscrew sliding technique resulted in notable vertical changes in craniofacial hard tissues. These changes included maxillary first molar intrusion, significant extrusion of maxillary central incisors, and a slight counterclockwise rotation of the mandibular plane.

## Data Availability

The data that support the findings of this study are available from the corresponding author upon reasonable request.
